# Cryogenic Tensile Strength of 1.6 GPa in a Precipitation-Hardened (NiCoCr)_99.25_C_0.75_ Medium-Entropy Alloy Fabricated via Laser Powder Bed Fusion

**DOI:** 10.3390/ma18153656

**Published:** 2025-08-04

**Authors:** So-Yeon Park, Young-Kyun Kim, Hyoung Seop Kim, Kee-Ahn Lee

**Affiliations:** 1 Department of Materials Science and Engineering, Inha University, Incheon 22212, Republic of Korea; soyeonpark@inha.edu; 2 Korea Institute of Materials Science (KIMS), Changwon 51508, Republic of Korea; ykkim@kims.re.kr; 3 Department of Materials Science and Engineering, Pohang University of Science and Technology, Pohang 37673, Republic of Korea; hskim@postech.ac.kr

**Keywords:** laser powder bed fusion, NiCoCr medium-entropy alloy, microstructure, nano-sized carbide, cryogenic mechanical property

## Abstract

A (NiCoCr)_99.25_C_0.75_ medium entropy alloy (MEA) was developed via laser powder bed fusion (LPBF) using pre-alloyed powder feedstock containing 0.75 at%C, followed by a precipitation heat treatment. The as-built alloy exhibited high density (>99.9%), columnar grains, fine substructures, and strong <111> texture. Heat treatment at 700 °C for 1 h promoted the precipitation of Cr-rich carbides (Cr_23_C_6_) along grain and substructure boundaries, which stabilized the microstructure through Zener pinning and the consumption of carbon from the matrix. The heat-treated alloy achieved excellent cryogenic tensile properties at 77 K, with a yield strength of 1230 MPa and an ultimate tensile strength of 1.6 GPa. Compared to previously reported LPBF-built NiCoCr-based MEAs, this alloy exhibited superior strength at both room and cryogenic temperatures, indicating its potential for structural applications in extreme environments. Deformation mechanisms at cryogenic temperature revealed abundant deformation twinning, stacking faults, and strong dislocation–precipitate interactions. These features contributed to dislocation locking, resulting in a work hardening rate higher than that observed at room temperature. This study demonstrates that carbon addition and heat treatment can effectively tune the stacking fault energy and stabilize substructures, leading to enhanced cryogenic mechanical performance of LPBF-built NiCoCr MEAs.

## 1. Introduction

Owing to their core effects—namely the high entropy effect, sluggish diffusion kinetics, cocktail effect, and severe lattice distortion—HEAs (high entropy alloys) can exhibit superior physical and chemical properties compared to conventional alloys [1,2,3,4]. Among them, the equiatomic CrMnFeCoNi alloy, often referred to as the Cantor alloy, is one of the earliest and most representative HEAs [4]. Due to its low stacking fault energy (SFE), this alloy exhibits deformation twinning (DT), which contributes to a favorable combination of strength and ductility from room to cryogenic temperatures [5,6]. Additionally, it has garnered significant interest due to its resistance to hydrogen embrittlement and its reasonable corrosion resistance [7,8,9,10].

Recently, there has been a growing demand across various industries—such as energy, aerospace, and polar engineering—for structural materials that maintain stable mechanical properties and environmental resistance over a wide temperature range, from cryogenic to high temperatures. In response to such extreme service environments, the Cantor HEA and its derivative alloys have shown promising potential in multiple aspects, including excellent oxidation resistance, radiation tolerance, and cryogenic toughness [11,12,13,14,15,16,17,18,19,20,21]. Against this backdrop, compositionally modified variants based on the Cantor HEA have been explored to tailor alloy properties, and research on MPEAs (multi-principal element alloys) has expanded rapidly in recent years [11,12,13,14,15,16,17,18,19,20,21].

Among the equiatomic MPEAs derived from the Cantor HEA, the NiCoCr medium entropy alloy (MEA) exhibits the most favorable mechanical properties over a wide temperature range, while also offering excellent oxidation resistance [15,16,17,18,19,20,21]. This alloy possesses the highest lattice friction stress and a low SFE below 22 mJ/m^2^ among Cantor-type alloys, which contributes to its notable fracture toughness, particularly at cryogenic temperatures [17,18,20]. However, its mechanical performance is highly sensitive to grain size, and achieving compositional homogeneity and refined grain structures typically requires complex thermo-mechanical processing routes [17,21,22].

To overcome these limitations, the implementation of laser powder bed fusion (LPBF) has gained increasing attention. This process offers the significant advantage of producing optimized parts with high geometrical freedom and precision. In particular, it has been proposed as an innovative manufacturing method that enables both compositional uniformity in MPEAs such as NiCoCr MEA and effective enhancement of mechanical properties [23,24,25,26,27,28,29,30]. The formation of strengthening particles such as oxides and the generation of high initial dislocation density during processing further contribute to strengthening, resulting in superior yield strength compared to alloys fabricated by conventional methods such as casting or rolling [23,24,25,26,27,28,29,30]. Leveraging these metallurgical benefits, various LPBF-based strategies have been proposed to improve the properties of NiCoCr MEA-based materials [23,24,29,30].

More recently, strategies have focused on incorporating strengthening particles into the NiCoCr MEA matrix by leveraging the characteristics of the LPBF process. Pan et al. [29] and Ma et al. [31] fabricated LPBF-built composites by blending TiC particles with NiCoCr MEA powder, achieving enhanced strength at both room and elevated temperatures. Hou et al. [32] mixed B_4_C precursor particles with NiCoCr MEA powder, leading to in situ formation of borides and carbides during processing, which resulted in favorable room-temperature mechanical properties. Ji et al. [33] employed a pre-alloying approach by introducing Al and Ti into the NiCoCr MEA composition, and subsequent post-heat treatment promoted the formation of Ni_3_(Al,Ti) precipitates. This precipitate, exhibiting good coherence with the matrix, contributed to improved strength at both room and high temperatures [33,34]. These approaches can be broadly categorized into mechanical milling and pre-alloying routes. The former often increases process complexity and cost due to additional powder preparation steps and may also induce undesirable particle degradation during processing [29,30,31,32]. The latter requires precise heat treatment control to induce precipitation and has been associated with the inevitable formation of Ti-, Al-, or Cr-rich oxides [33]. The mechanical properties of the resulting alloys have been primarily reported at room and elevated temperatures, while cryogenic performance has only been discussed in the context of TiC addition [31].

In previous studies, the present authors employed a strategy in which 0.25 or 0.75 at.% carbon was pre-alloyed into the NiCoCr MEA, followed by LPBF processing to promote in situ formation of strengthening particles through reactions between carbon (and partially oxygen) and Cr [35]. (NiCoCr)_99.25_C_0.75_ MEA containing 0.75 at.% carbon exhibited higher oxidation resistance and superior room-temperature strength compared to its cast counterpart [35,36]. Furthermore, post-heat treatment was applied to stabilize the matrix and induce additional carbide precipitation, resulting in enhanced mechanical properties at both room and elevated temperatures [37,38].

In this study, a precipitation strengthening heat treatment was applied to a NiCoCr MEA with 0.75 at.% C fabricated via LPBF. Cryogenic tensile tests were conducted, and the microstructures of specimens before and after deformation were analyzed. Based on the resulting mechanical performance at cryogenic temperatures, this study aimed to explore the potential of LPBF-built (NiCoCr)_99.25_C_0.75_ MEA as a next-generation structural material for extreme environments.

## 2. Experimental Methods

### Material Preparation

Carbon was added to an equiatomic NiCoCr melt to achieve an atomic concentration of 0.75 at.%, and the alloy was solidified to produce a pre-alloyed NiCoCr MEA ingot. Powder feedstock was subsequently fabricated from the ingot via gas atomization. The produced powder was sieved to obtain particles smaller than approximately 50 μm. The selected powder exhibited a d_50_ of 25.9 μm and a spherical morphology, making it suitable for application in the LPBF process.

**Figure 1:**
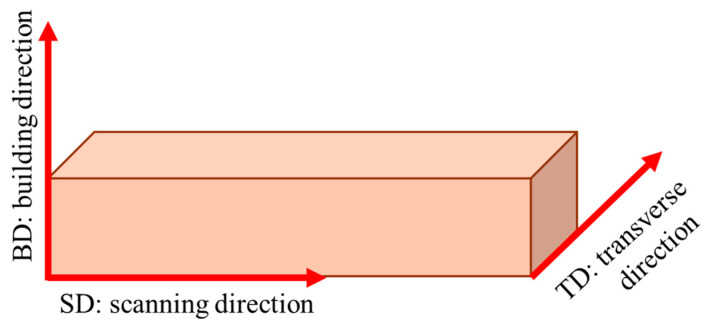
Schematic diagram indicating information regarding the specimen’s direction.

The LPBF process was performed using an MCP HEK REALIZER system (SLM Solutions GmbH, Lübeck, Germany), and the optimized processing parameters were adopted from previous work [35]. The process conditions were set as follows: a scan speed of 600 mm/s, hatch spacing of 0.08 mm with 30% overlap, laser power of 90 W, beam diameter of 0.11 mm, and layer thickness of 25 μm. The orientation of the fabricated specimens was defined as scanning direction (SD), building direction (BD), and transverse direction (TD), as illustrated in Figure 1. The chemical composition of the fabricated alloy was Ni: 30.12 at.%, Co: 31.31 at.%, and Cr: 37.53 at.%, with a carbon content of 0.75 at.%, consistent with the amount added to the ingot. The alloy in the as-built condition is hereafter referred to as 0.75C MEA.

To increase the precipitate fraction in the fabricated 0.75C MEA, peak aging conditions (700 °C for 1 h followed by water quenching), derived from previous work [37], were applied. The heat treatment was carried out under ambient atmosphere. The peak aging condition was determined by fixing the holding time at 1 h and varying the treatment temperature from 600 to 800 °C in 50 °C intervals, selecting the condition that yielded the highest Vickers hardness. The specimen subjected to this heat treatment is hereafter referred to as 0.75C MEA HT.

X-ray diffraction (XRD, X’Pert Pro MRD, PANalytical, Coventry, UK) was conducted to identify the constituent phases and determine the lattice parameter of the LPBF-built MEAs. Samples for microstructural analysis were sectioned from the central region of specimens using a high-speed rotary cutter equipped with a ceramic blade, followed by grinding and mirror polishing. Surface preparation involved sequential grinding with 1200–4000 grit silicon carbide papers, polishing with a 1 μm diamond suspension, and final polishing with 0.04 μm colloidal silica. Microstructural observations were performed using field-emission scanning electron microscopy (FE-SEM, MYRA3 XMH, TESCAN, Brno, Czech Republic) and electron backscatter diffraction (EBSD, Nordlys Nano detector, Oxford Instruments, Abingdon, UK) attached to the FE-SEM. EBSD analysis was conducted with a step size of 0.8 μm and an accelerating voltage of 15 kV. The acquired data were processed using OIM Analysis software (TSL OIM Analysis 8, EDAX, Mahwah, NJ, USA).

Cryogenic tensile testing of 0.75C MEA HT was conducted using a universal testing machine (INSTRON 5982, Instron, a division of Illinois Tool Works, Inc., Glenview, IL, USA), with standard specimens having a gauge length of 30 mm and a gauge diameter of 6.5 mm. The test was performed along the scanning direction (SD) at an initial strain rate of 10^−3^ s^−1^. A thermally insulated chamber mounted on the tensile machine was used to maintain the test temperature. Liquid nitrogen was continuously supplied to the chamber to sustain the equilibrium temperature of liquid nitrogen (77 K). Each mechanical test was performed three times, and the mean value of the results was adopted as the representative value.

After the cryogenic tensile test, fractography was carried out using field-emission scanning electron microscopy (FE-SEM, MYRA3 XMH). For microstructural observation of the deformed specimens, the cross-sections were prepared using the same grinding and polishing procedures as those used for the initial microstructural analysis. FE-SEM and EBSD analyses were performed, with EBSD conducted at a step size of 0.3 μm and an accelerating voltage of 15 kV.

## 3. Results and Discussion

### 3.1. Microstructure Observation and Analysis Results

**Figure 2:**
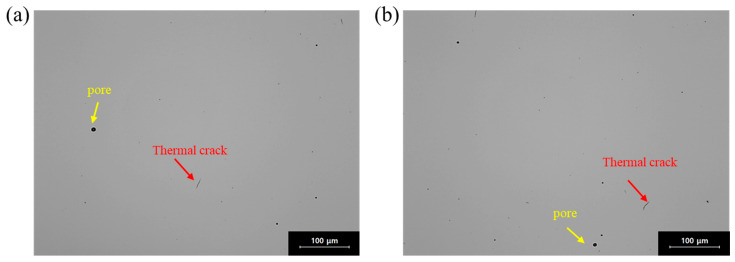
Optical microscopic images showing distribution of defects in LPBF-built 0.75C MEA on each plane: (**a**) BD plane and (**b**) SD plane.

Figure 2 presents optical microscopy (OM) images showing defect distribution in the 0.75C MEA. Using an image analyzer (ImageJ Pro, NIH, Bethesda, MD, USA), the average porosity was measured to be 0.08 ± 0.03% on the BD plane and 0.10 ± 0.04% on the SD plane, indicating an excellent relative density of approximately 99.9%. Lack-of-fusion defects were rarely observed, while a small number of thermal cracks and gas-trapped pores were present.

**Figure 3:**
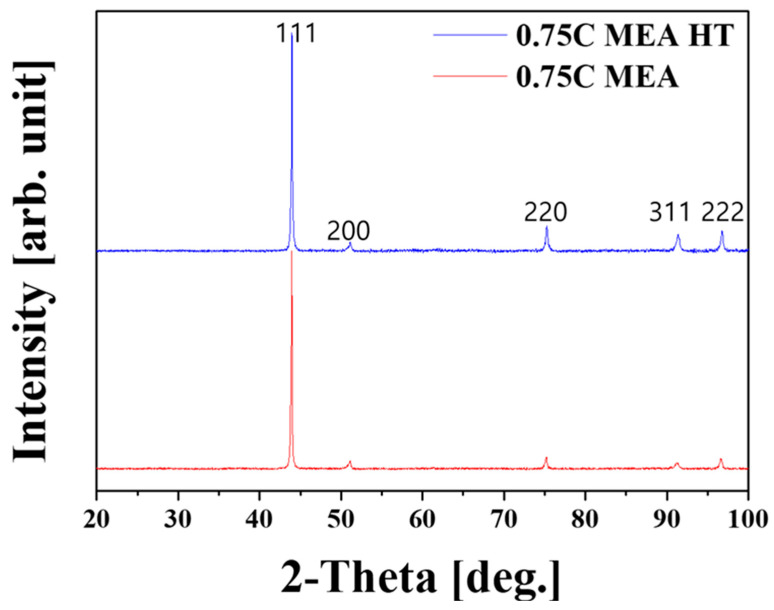
XRD analysis results of 0.75C MEA HT and 0.75C MEA.

Figure 3 shows the XRD analysis results for 0.75C MEA and 0.75C MEA HT. In both conditions, only peaks corresponding to a face-centered cubic (FCC) single phase were detected, and peaks from other phases such as carbides were not clearly distinguishable due to their weak intensity. Based on the diffraction peaks, lattice constants were calculated using Bragg’s law. A slight contraction of the lattice was detected, from 3.568 Å in the as-built state to 3.571 Å after heat treatment. This result suggests that carbon atoms, which were initially dissolved interstitially in the FCC matrix, were consumed during carbide precipitation upon heat treatment [39,40].

**Figure 4:**
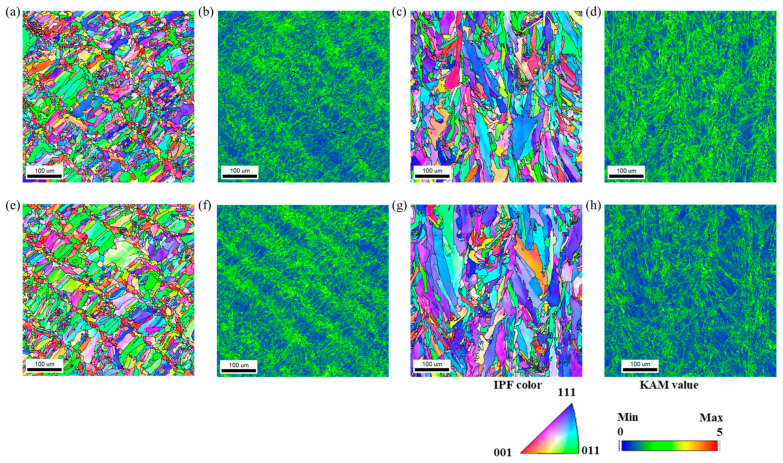
EBSD IPF maps and KAM maps of LPBF-built 0.75C MEA and 0.75C MEA HT. IPF map of the 0.75C MEA (**a**) BD plane with (**b**) a KAM map on the BD plane, and (**c**) the SD plane with (**d**) a KAM map on the SD plane, and an IPF map of the 0.75C MEA HT (**e**) BD plane with (**f**) a KAM map on the BD plane, and (**g**) the SD plane with (**h**) a KAM map on the SD plane.

Figure 4 presents EBSD maps of 0.75C MEA and 0.75C MEA HT taken from the BD plane (perpendicular to the building direction) and the SD plane (perpendicular to the scanning direction). The difference in microstructures between the BD and SD planes is commonly observed in alloys fabricated by the LPBF process, and this phenomenon is also frequently reported in HEAs and MEAs [27,28,29,30]. This behavior follows the mechanism in which a semi-elliptical molten pool is formed by laser irradiation during the LPBF process, followed by rapid solidification from the bottom of the molten pool. In the BD plane, both alloys exhibited epitaxial grain growth morphology along the BD direction, regardless of the heat treatment, and were composed of columnar grains nearly aligned with the BD. In the SD plane, ultrafine grains were observed along the laser scan tracks, surrounded by relatively coarser grains, indicating microstructural heterogeneity. 0.75C MEA also exhibited strong <111> and <011> textures along the BD direction, which were retained after heat treatment. Gradient color distributions within individual grains were visible in both the as-built and heat-treated specimens, suggesting the presence of developed substructures. The average grain sizes were measured to be 9.07 μm (BD) and 14.01 μm (SD) for 0.75C MEA and 9.23 μm (BD) and 14.76 μm (SD) for 0.75C MEA HT, indicating minimal grain growth after heat treatment. Misorientation angles were categorized as follows: boundaries with angles greater than 15° were defined as high-angle grain boundaries (HAGBs), while those between 5° and 15° were classified as low-angle grain boundaries (LAGBs). The fraction of HAGBs showed little change, increasing slightly from 36.0% in the as-built condition to 36.5% after heat treatment. In contrast, the fraction of LAGBs increased from 5.6% to 10.3% following heat treatment. Overall, these results suggest that grain-level microstructural evolution involving high-angle boundaries was limited during heat treatment, with the fine-grained structure and heterogeneity largely preserved. However, the increase in LAGB fraction implies that dislocation structures with misorientation angles below 5° may have undergone structural rearrangement during the heat treatment process.

**Figure 5:**
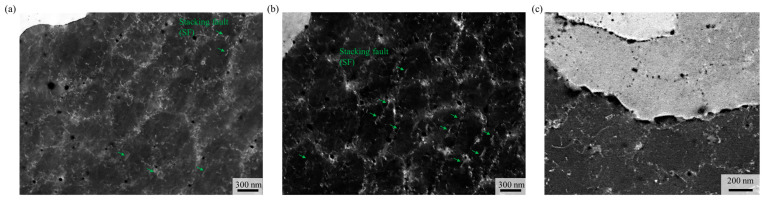
ECC images showing the dislocation, stacking faults (SF), and precipitates of (**a**) 0.75C MEA and (**b**,**c**) 0.75C MEA HT.

In contrast, the KAM map analyses on the SD plane (Figure 4d,h) revealed changes in dislocation structures after heat treatment. Under both conditions, low-angle misorientation boundaries (<5°) were present within grains, indicating the formation of columnar or cellular substructures associated with dislocations. Prior to heat treatment, the substructure boundaries—as well as the grain interiors—exhibited generally high and uniform KAM values. The geometrically necessary dislocation (GND) density, calculated from EBSD data, was as high as 12.7 × 10^13^/m^2^. After heat treatment, although the overall dislocation density decreased slightly, the substructure morphology was largely retained. The GND value was reduced to approximately 6.2 × 10^13^/m^2^, indicating that the dislocation density decreased by about half following heat treatment. On the other hand, the KAM map analyses on the BD plane (Figure 4b,f) did not reveal a significant difference in KAM values. This may be attributed to the relatively high fraction of ultrafine grains present on the BD plane, where precipitates formed along grain boundaries (Figure 5) are likely to exert a Zener pinning effect.

To further investigate the distribution of dislocations and precipitates in 0.75C MEA and 0.75C MEA HT, electron channeling contrast imaging (ECCI) and transmission electron microscopy (TEM) were performed, and the results are shown in Figure 5. Cellular substructures with characteristic dimensions of several hundred nanometers were observed within the grains. These substructures appeared as dislocation networks bounded by tangled dislocations. Such features are commonly observed in alloys fabricated by LPBF and are attributed to the repeated thermal cycling and rapid cooling rates (on the order of 10^5^–10^7^ K/s) during the process, which lead to partial plastic deformation and the accumulation of dislocations within cells [41]. Following heat treatment, the dislocations concentrated along the substructure boundaries were partially recovered, and many of the dislocations initially present within the substructure interiors were relaxed. As a result, a moderate reduction in overall dislocation density was observed.

**Figure 6:**
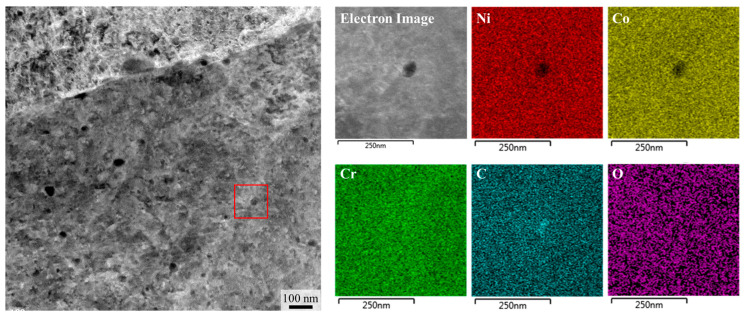
TEM-EDS results showing the distribution of precipitates and their chemical composition in 0.75C MEA HT.

Meanwhile, ECC image analyses before and after heat treatment revealed a noticeable increase in both the number and volume fraction of dark precipitate particles after heat treatment. These particles were identified via TEM-EDS (Figure 6) as Cr-rich carbides, and based on previous studies [35,36,37,38] they were classified as Cr_23_C_6_ carbides with partial inclusion of oxides. After heat treatment, coarse carbides approximately 200 nm in size were observed along grain boundaries, while numerous fine Cr_23_C_6_ carbides ranging from a few to several tens of nanometers precipitated along substructure boundaries. The overall carbide volume fraction was estimated to be approximately 2.25%. A strong contrast at the interface between the carbides and the matrix was observed in the ECC images, indicating that dislocations were highly concentrated around the carbides due to their pinning effect. In conventionally processed NiCoCr MEAs, rapid recrystallization is known to occur at temperatures above 600 °C, leading to deterioration in mechanical properties [42]. In contrast, the present study showed that the substructures remained intact, and grain size was largely preserved, after heat treatment. This behavior is attributed to the Zener pinning effect of the carbides, which likely suppressed grain boundary migration and dislocation movement [40].

In addition, 0.75C MEA exhibited an increased fraction of stacking faults (SFs) after heat treatment. NiCoCr MEA is inherently known to have a low stacking fault energy (SFE) of approximately 18 mJ/m^2^, which facilitates the formation of SFs [43]. However, carbon dissolution in the matrix is reported to increase the SFE; for instance, the addition of 0.75 at.% carbon has been reported to raise the SFE by approximately 7 mJ/m^2^ [18]. Therefore, prior to heat treatment, the SFE was likely elevated due to carbon dissolution in the FCC matrix. During heat treatment, however, carbon was consumed via carbide precipitation, leading to a reduction in SFE. This decrease in SFE after heat treatment is believed to have promoted the formation of SFs, resulting in the higher fraction of SFs observed in 0.75C MEA HT.

### 3.2. Cryogenic Tensile Mechanical Property

**Figure 7:**
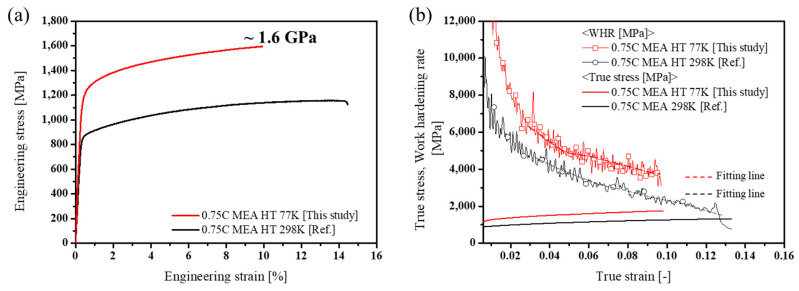
The results of cryogenic and room temperature [37] tensile tests of 0.75C MEA HT: (**a**) engineering stress–strain curves and (**b**) work hardening rate–strain curves with true stress–strain curves.

Figure 7a presents the tensile stress–strain curves of 0.75C MEA HT tested at both cryogenic and room temperatures, and the corresponding mechanical properties are summarized in Table 1. At 77 K, the alloy exhibited a yield strength (σ_y_) of 1230.0 MPa, an ultimate tensile strength (UTS) of 1595.1 MPa, and a fracture elongation (ε_f_) of 9.4%. Compared to the room-temperature properties reported in a previous study [37] (σ_y_ = 872.7 MPa, UTS = 1156.9 MPa, and ε_f_ = 14.5%), the cryogenic yield and ultimate strengths increased by 357.3 MPa and 438.2 MPa, respectively, while the elongation decreased.

**Figure 8:**
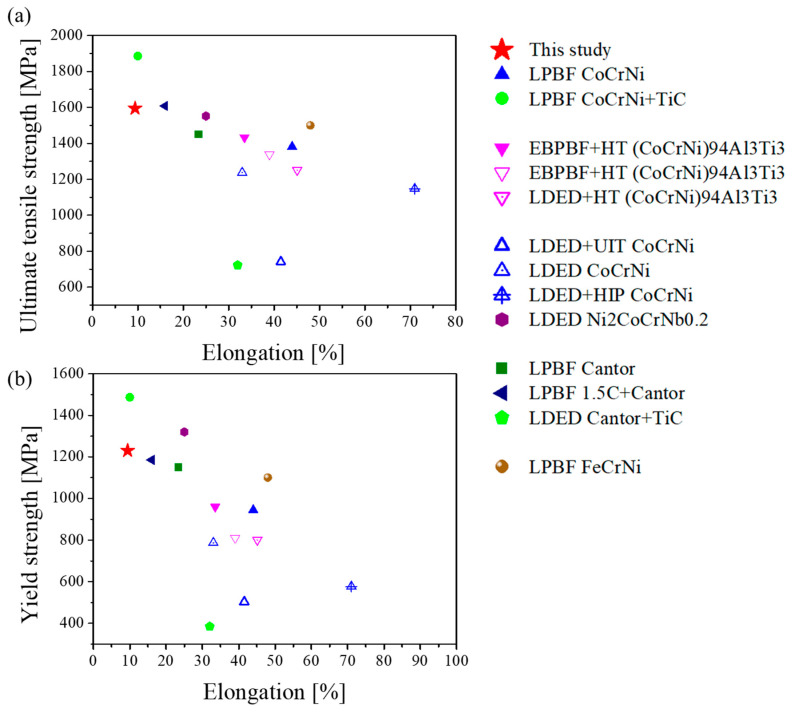
Comparison of tensile mechanical properties at 77 K for various HEAs and MEAs fabricated by different additive manufacturing processes [31,44,45,46,47,48,49,50,51,52,53]. Abbreviations: LDED, Laser Directed Energy Deposition; EBPBF, Electron Beam Powder Bed Fusion; UIT, Ultrasonic Impact Treatment; HIP, Hot Isostatic Pressing.

The cryogenic tensile properties obtained in this study were compared with those of various HEAs and MEAs fabricated via additive manufacturing processes at 77 K, as summarized in Figure 8 [31,44,45,46,47,48,49,50,51,52,53]. The ultimate tensile strength (UTS) of 0.75C MEA subjected to heat treatment (HT) in this study was comparable to that of the LPBF-processed 1.5 at.%C Cantor alloy previously reported by our group [51], while its yield strength was higher. Among the reported AM-fabricated HEAs/MEAs, CoCrNi MEA with 4 wt.% TiC addition exhibited the highest strength values [31]. However, this approach requires a ball milling process for TiC addition, and during LPBF processing, TiC particles tend to float to the top of the melt pool, leading to partially inhomogeneous distribution. Overall, 0.75C MEA HT demonstrated a UTS of 1.6 GPa, placing it among the top two to three strongest AM-fabricated MEAs/HEAs reported to date. In comparison, LPBF-built 316L stainless steel—a representative cryogenic structural material—exhibits a UTS of 1083 MPa at 77 K [54]. These findings suggest that 0.75C MEA HT offers outstanding mechanical properties, highlighting its strong potential for application as a cryogenic structural material.

Work hardening rate (WHR) versus true strain curves, derived from the true stress–strain data, are shown in Figure 7b. The results indicate that the WHR at cryogenic temperature consistently remained approximately 1 GPa higher than that at room temperature across the entire strain range, demonstrating enhanced work hardening capability. Furthermore, the slope of the WHR curve decreased more gradually at cryogenic temperature than at room temperature, suggesting that additional hardening mechanisms may be active at low temperatures. In Cantor-type HEAs or MEAs such as NiCoCr MEA, the increase in WHR at cryogenic temperature is generally attributed to a reduction in SFE with decreasing temperature, which promotes a higher frequency and fraction of deformation twinning (TWIP effect) [55,56]. Therefore, the observed WHR behavior is likely related to temperature-dependent changes in SFE.

### 3.3. Fracture and Deformation Mechanisms

**Figure 9:**
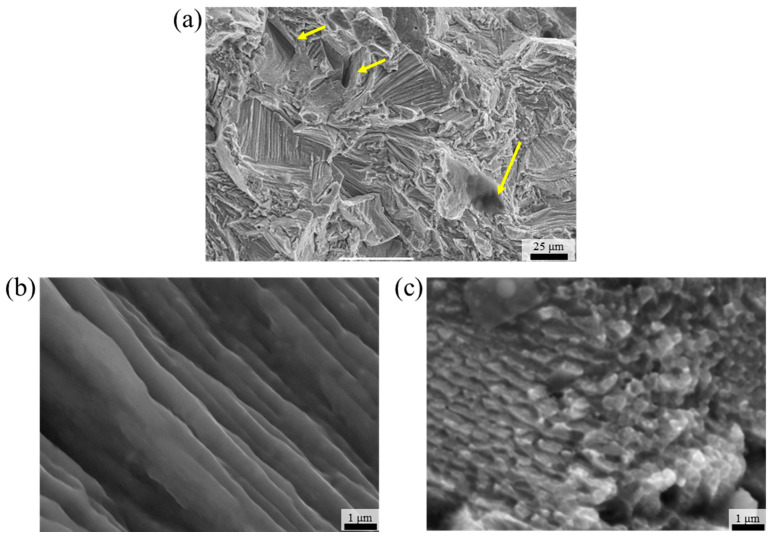
SEM fractography observation results for 0.75C MEA HT after a cryogenic tensile test: (**a**) low-magnification image and morphologies of the (**b**) columnar substructure and (**c**) cellular substructure. The yellow arrows indicate traces of pre-existing defects.

Figure 9 shows the SEM fractography results used to analyze the cryogenic fracture mechanism of the 0.75C MEA HT specimen. The alloy exhibited features characteristic of a quasi-cleavage fracture mode at 77 K. Tear ridges were formed along individual grains, and crack propagation was observed to occur along grain boundaries. In some regions, signs of fracture initiation at pre-existing defects such as cracks and pores (indicated by the yellow arrows in Figure 9a) were observed. These initial defects, which are commonly found in materials fabricated by additive manufacturing, contribute to the reduction in elongation. High-magnification images revealed exposed columnar and cellular structures on the fracture surface, indicating that the substructures likely served as initiation and propagation sites for cracking. As reported in a previous study [37], partial dimples containing carbides were observed at 298 K, indicating some degree of ductile fracture. In contrast, such dimples were scarcely observed at 77 K, suggesting reduced ductility under cryogenic conditions. These results imply that, while the nanoscale precipitates in 0.75C MEA HT contribute to strength enhancement via Orowan strengthening, they may also serve as crack nucleation sites at cryogenic temperatures, thereby reducing elongation.

**Figure 10:**
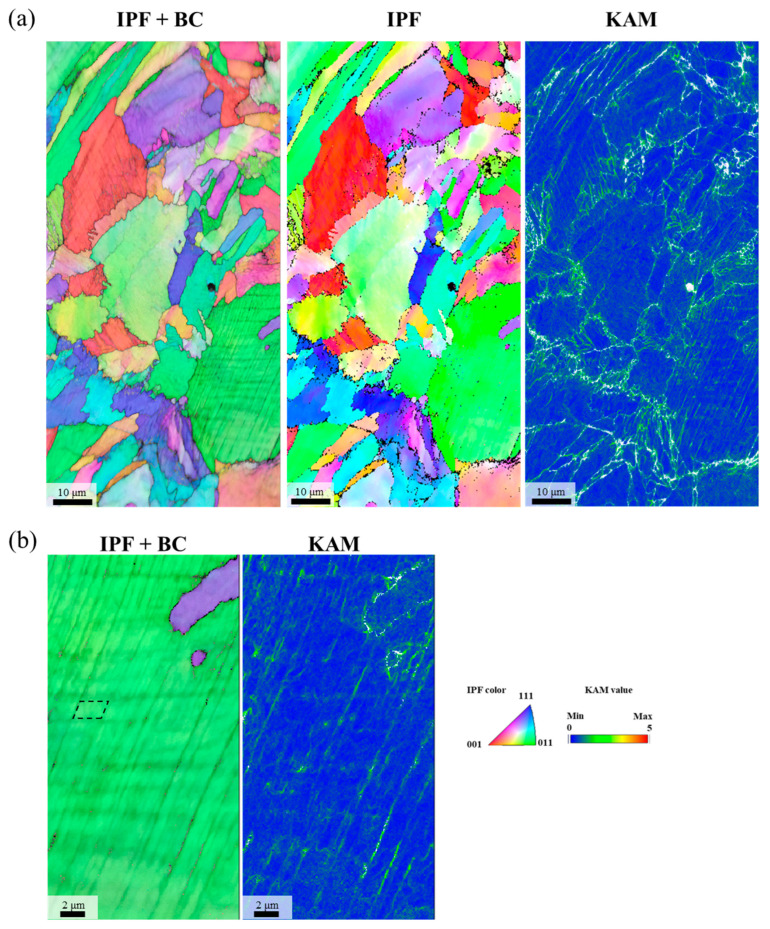
EBSD analysis results showing the cross-sectional microstructure of 0.75C MEA HT after a cryogenic tensile test: (**a**) low magnification and (**b**) high magnification. The rectangular in (**b**) shows intersecting parallel lines forming parallelogram-shaped domains within a grain.

**Figure 11:**
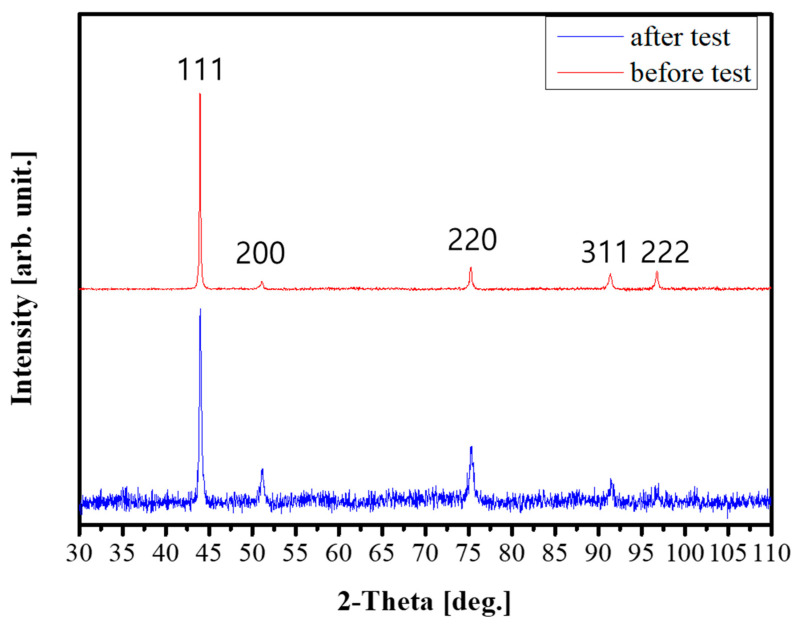
XRD analysis results for 0.75C MEA HT before and after cryogenic deformation.

Figure 10 presents EBSD maps of the cross-sectional microstructure beneath the fracture surface of the 0.75C MEA HT specimen after a cryogenic tensile test. The grain boundary regions exhibited elevated KAM values, suggesting a strong strengthening effect associated with grain boundary activity. The grains appeared serrated in shape, and carbide precipitates located at the grain boundaries were observed to exert a pinning effect, likely suppressing grain boundary migration and contributing to microstructural stability. Subgrain structures within individual grains also became more clearly defined after deformation. In some grains, a high density of parallel lines was observed, and intersecting lines in two directions formed parallelogram-like domains. These intersections exhibited pronounced local strain accumulation, indicating significant intragranular deformation. Comparison of the XRD profiles before and after deformation (Figure 11) showed an increase in the relative intensities of the [200] and [220] peaks, indicating texture evolution during deformation and suggesting the occurrence of substantial deformation twinning. No evidence of a phase transformation to a hexagonal close-packed (HCP) structure was observed, and the matrix retained its FCC single-phase structure.

**Figure 12:**
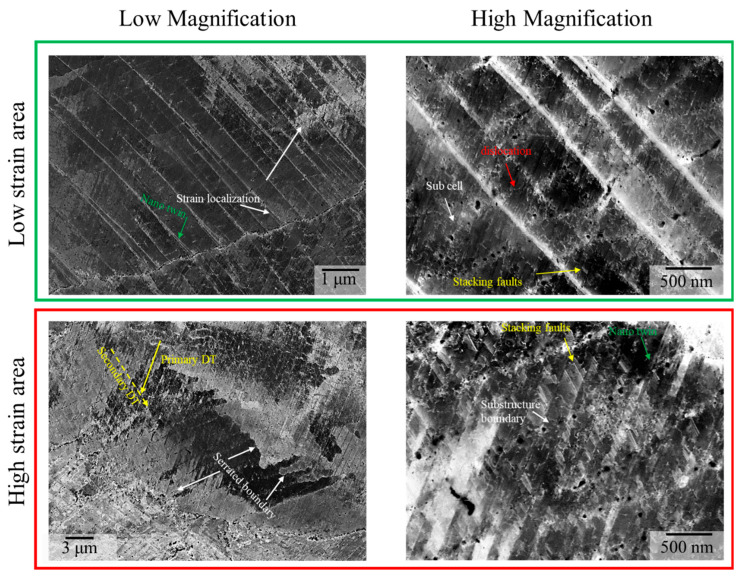
ECC images of cryogenically deformed 0.75C MEA HT showing deformation twins, stacking faults, and strain localization across low- and high-strain regions.

To closely examine the morphology and distribution of metallurgical defects such as dislocations, stacking faults (SFs), and twins after deformation, ECC image analysis was conducted on the cryogenically deformed specimen. The results are shown in Figure 12. A notable feature of the cryogenically deformed sample was the abundant formation of deformation twins (DTs). In contrast, as reported in a previous study [37], ECC images of specimens subjected to room-temperature tensile testing showed that strengthening primarily arose from dislocation motion suppression by refined substructures, with SFs contributing to the enhanced work hardening rate, while no evidence of DTs was observed. This absence of twinning at room temperature, despite low SFE, has been attributed to the suppression of DT evolution when the spacing between precipitates is narrow [51,57]. In the cryogenically deformed specimen, however, numerous nano-scale twin boundaries exhibiting strong contrast against the matrix were observed in ECC images. Unlike at room temperature, where DTs were absent even in high-strain regions, cryogenic conditions promoted the formation of multiple DTs across substructures even in low-strain regions. This behavior is likely due to the further reduction in SFE at low temperatures [56,58]. Simultaneously, dislocations were observed to accumulate along substructure boundaries in low-strain regions, contributing to substructure stabilization, along with the formation of a small number of SFs. Significant strain localization was observed in regions where DTs intersected with substructures and high-angle grain boundaries. These intersections likely promoted dislocation locking and contributed substantially to strengthening of the alloy.

In the high-strain regions, low-magnification ECC images revealed collective evolution of DTs. Both primary and secondary DTs were observed, intersecting at an angle of approximately 60°, indicative of multi-directional twin formation. Additionally, EBSD analysis revealed the presence of serrated grain boundaries. These boundaries were observed not only along high-angle grain boundaries containing relatively coarse precipitates in the initial microstructure, but also along regions that originally corresponded to substructure boundaries. In the latter case, it is likely that dislocations accumulated at precipitate-pinned substructure boundaries, gradually increasing local misorientation and resulting in transformation into serrated grain boundaries.

High-magnification ECC images of the high-strain region clearly demonstrate the strengthening mechanisms operative during deformation: (1) a high density of nano-scale DTs, (2) numerous SFs, and (3) substructures stabilized by precipitates. These features intersected and served as effective barriers to dislocation motion. Acting similarly to grain boundaries during deformation, these intersecting features likely gave rise to a dynamic Hall–Petch effect, which contributed to uniform plastic deformation and enhanced work hardening. Notably, the extensive formation of DT boundaries and SFs at cryogenic temperature promotes the operation of Lomer lock and Hirth lock mechanisms in FCC metals, which can contribute to strength enhancement [18,57,59]. According to Kumar et al. [44], LPBF-processed CoCrNi alloys exhibited limited DT evolution even at 77 K, which was attributed to an increased SFE caused by interstitial solutes such as O and C. In contrast, in the present study, the heat treatment applied to 0.75C MEA HT likely reduced the SFE by consuming interstitial atoms initially dissolved in the matrix. This reduction in SFE may have promoted DT and SF evolution, ultimately contributing to the high tensile strength that was observed.

Although the alloy developed in this study achieved high strength, it exhibited relatively low elongation. Several factors may have contributed to this outcome. First, the carbide precipitates used as strengthening agents are likely to have low lattice coherence with the matrix. While such particles can effectively enhance strength via the Orowan mechanism, they may also adversely affect ductility. In addition, the alloy contains a high volume fraction of densely distributed nano-scale deformation twins and stacking faults (SFs). As discussed earlier, these features contribute to an increased work-hardening rate and high tensile strength, but they are also reported to significantly hinder dislocation glide during deformation, thereby limiting work-hardening capacity [60]. Moreover, internal defects that are inevitably introduced during the additive manufacturing process may further reduce elongation, particularly under cryogenic conditions. Therefore, future alloy design may benefit from considering strategies such as the use of coherent precipitates or interfacial control, post-processing heat treatments to mitigate defects, and tailoring the stacking fault energy (SFE) to control the volume fraction of twins and SFs, with the aim of improving both strength and ductility.

## 4. Conclusions

In this study, a precipitation-strengthened NiCoCr medium entropy alloy (MEA) was fabricated via laser powder bed fusion (LPBF) using pre-alloyed (NiCoCr)_99.25_C_0.75_ powder, followed by precipitation heat treatment. The microstructural features and cryogenic tensile properties of the alloy were systematically investigated. The main conclusions are as follows:

1. The 0.75C MEA fabricated via LPBF exhibited a high relative density (>99.9%), columnar grain structure aligned along the building direction (BD), and refined substructures, all of which were retained after heat treatment. Post-heat treatment at 700 °C promoted the precipitation of Cr-rich carbides (Cr_23_C_6_) along grain and substructure boundaries. These precipitates contributed to microstructural stabilization by suppressing grain growth and dislocation motion via the Zener pinning effect. High dislocation densities were observed both before and after heat treatment, with the average dislocation density decreasing to approximately 6.2 × 10^13^ m^−2^ after heat treatment. Additionally, the fraction of stacking faults increased after heat treatment, likely due to a reduction in carbon solubility in the matrix, which decreased the stacking fault energy.
2. Heat-treated 0.75C MEA exhibited excellent mechanical properties at cryogenic temperature (77 K), with a yield strength of 1.23 GPa, an ultimate tensile strength of 1.60 GPa, and an elongation of 9.4%. These values position it among the top two to three highest strength levels reported for AM-fabricated MEAs and HEAs to date. Notably, its ultimate tensile strength surpasses those of previously reported PBF-built precipitation-strengthened NiCoCr MEA. Compared to room temperature, the alloy demonstrated higher yield and tensile strengths at 77 K, accompanied by a reduction in elongation.
3. Post-deformation microstructural analysis revealed that extensive deformation twinning (DT) occurred under cryogenic conditions, which was attributed to the reduced stacking fault energy (SFE). DTs intersected with high-angle grain boundaries and substructure boundaries, leading to dislocation locking and the activation of a dynamic Hall–Petch effect, resulting in a high work hardening rate (WHR). While dislocation restriction by substructures and stacking faults was the dominant strengthening mechanism at room temperature, at 77 K, the combined interaction among DTs, SFs, and precipitates acted as a synergistic strengthening mechanism, contributing significantly to both uniform deformation and high strength retention. Furthermore, WHR vs. true strain analysis showed that the WHR remained consistently higher—by approximately 1 GPa—across the entire strain range at cryogenic temperature. These findings suggest that multiple temperature-sensitive strengthening mechanisms were sequentially activated in this alloy, enabling the simultaneous retention of high strength and superior work hardening capability under cryogenic conditions.
4. In this study, a NiCoCr MEA alloy with excellent cryogenic tensile strength was successfully developed by employing the LPBF process in combination with carbide strengthening. For future work, it will be necessary to evaluate the material’s potential as a next-generation structural alloy operable over a wide temperature range by conducting additional tests such as fatigue, creep, and fracture toughness.

## Figures and Tables

**Table 1:** Tensile properties at cryogenic and room temperatures. The room temperature data were taken from our previous study [37].

Temperature	Yield Strength [MPa]	Ultimate Tensile Strength [MPa]	Fracture Elongation [%]	
77 K	1230.0 ± 7.8	1595.1 ± 2.1	9.4 ± 1.6	Present
298 K	872.7	1156.9	14.5	[37]

## Data Availability

The original contributions presented in this study are included in the article. Further inquiries can be directed to the corresponding author.
